# Treetrimmer: a method for phylogenetic dataset size reduction

**DOI:** 10.1186/1756-0500-6-145

**Published:** 2013-04-12

**Authors:** Shinichiro Maruyama, Robert JM Eveleigh, John M Archibald

**Affiliations:** 1 Department of Biochemistry & Molecular Biology, Dalhousie University, Halifax, NS, Canada; 2 Centre for Comparative Genomics and Evolutionary Bioinformatics, Dalhousie University, Halifax, NS, Canada; 3 Integrated Microbial Biodiversity Program, Canadian Institute for Advanced Research, Montreal, QC H3A 1A4, Canada; 4 McGill University and Génome Québec, 740 Docteur-Penfield Ave, Montreal, QC H3A 1A4, Canada

**Keywords:** TreeTrimmer, Phylogenetic tree, Pruning, Dereplication, Taxonomic category

## Abstract

**Background:**

With rapid advances in genome sequencing and bioinformatics, it is now possible to generate phylogenetic trees containing thousands of operational taxonomic units (OTUs) from a wide range of organisms. However, use of rigorous tree-building methods on such large datasets is prohibitive and manual ‘pruning’ of sequence alignments is time consuming and raises concerns over reproducibility. There is a need for bioinformatic tools with which to objectively carry out such pruning procedures.

**Findings:**

Here we present ‘TreeTrimmer’, a bioinformatics procedure that removes unnecessary redundancy in large phylogenetic datasets, alleviating the size effect on more rigorous downstream analyses. The method identifies and removes user-defined ‘redundant’ sequences, e.g., orthologous sequences from closely related organisms and ‘recently’ evolved lineage-specific paralogs. Representative OTUs are retained for more rigorous re-analysis.

**Conclusions:**

TreeTrimmer reduces the OTU density of phylogenetic trees without sacrificing taxonomic diversity while retaining the original tree topology, thereby speeding up downstream computer-intensive analyses, e.g., Bayesian and maximum likelihood tree reconstructions, in a reproducible fashion.

## Background

Molecular phylogeny is a powerful tool for inferring evolutionary relationships. With advances in high-throughput genome and transcriptome sequencing it is now possible to construct trees from nucleic acid and protein sequence alignments containing thousands of OTUs. Despite the obvious potential for improving our understanding of the history of modern-day organisms and their genomes, an important downside of this ‘embarrassment of riches’ is the fact that many phylogenetic trees are produced using datasets that have been trimmed down to a ‘manageable’ size for methodological and/or presentation purposes [1,2].

A common adjustment is to reduce the dataset size to a level examinable by the human eye on a case-by-case basis. A large number of similarity search hits are often retrieved iteratively and sorted, with the user manually retaining sequences from taxa of interest along with a few arbitrarily chosen representatives from other ‘less important’ taxa. Alternatively, all sequences available with similarity scores above a certain threshold can be aligned and used to construct a ‘quick and dirty’ tree, based on which decisions are made about which sequences to keep and which to discard. A second tree within the desired number of OTUs is then constructed. Although such procedures are commonplace, they tend to be performed in an ad hoc, subjective fashion that is difficult to accurately reproduce. Here we present TreeTrimmer, a method for reducing the complexity and redundancy of phylogenetic trees by removal of phylogenetically and taxonomically closely related OTUs, with minimal loss of taxonomic diversity. Two useful applications of the method are discussed herein.

## Methods

### Phylogenetic tree construction

For tree constructions, homologous sequences were collected using BLASTP v2.2.26+ (option: -evalue 1e-5 -max_target_seqs 2000, unless otherwise specified in figure legends) [3], and aligned with MAFFT v6.864 (option: --auto --anysymbol) [4]. After trimming the multiple sequence alignments using trimAL v1.4 (option: -gt 0.8) [5], approximately-maximum-likelihood phylogenetic trees were constructed with fasttree v2.1.3 (option: -pseudo -spr 4 -mlacc 2 -slownne -wag -gamma) [6].

### The ‘TreeTrimmer’ procedure

**Figure 1:**
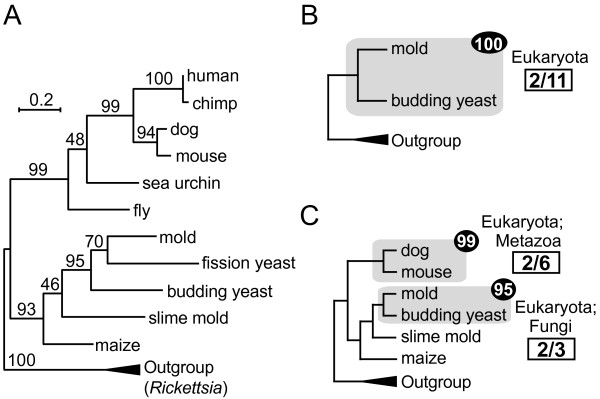
**Reduction of OTU complexity in phylogenetic trees with TreeTrimmer.** (**A**) A phylogenetic tree of mitochondrial Cytochrome c oxidase subunit 2 protein sequences with support values. (**B**) A trimmed reference tree (cladogram) derived from analysis of the tree shown in A, generated using the first rank of the taxonomic category ‘Eukaryota’ to assess whether all the OTUs belong to the same category in each highly supported clade. The extent of OTU reduction (i.e., dereplication) is shown in boxes. The number in the closed circle indicates the support value in the original tree. (**C**) Results showing the tree in A pruned with different parameter settings, in this case with the second rank of eukaryotic OTUs, i.e., a more ‘fine-scale’ evolutionary depth. As in B, the tree is shown as a cladogram.

TreeTrimmer is a command-line program written in Ruby, developed as part of a phylogenomic pipeline used in the analysis of algal nuclear genome sequences [7]. As input the program requires (i) a Newick formatted tree file with or without statistical support values (e.g., bootstrap values, Bayesian posterior probabilities; Figure 1) calculated by any phylogenetic method, (ii) a parameter input file (Additional file 1: Figure S1B, C) and (iii) optionally a reference list of OTU names with relevant taxonomic information (Additional file 1: Figure S1E). The parameter input file includes a user-defined support value threshold that TreeTrimmer uses to identify well-supported clades for ‘de-replication’. If the threshold is specified as ‘0’, all possible clades are examined. The parameter input file also includes user-defined information on how many OTUs are to be pruned or ‘de-replicated’ (i.e., trimmed down to a few representatives) for each clade (or subtree) for a given taxonomic category and at which taxonomic level (e.g., class, family, species, user-specified categories). Alternatively, instead of taxonomic category, other kinds of information (e.g., sample site names, geographical locations, project/version data) can be used for ‘de-replication’ in the context of metagenomic/environmental sequence analyses. The procedure works as described below.

First, based on the position of the root defined in the Newick tree file, the tree is examined for internal nodes associated with a support value equal to or greater than the user-defined threshold (or ‘highly supported clade’). For each such node, the method considers whether or not the OTUs contained in each of these highly supported clades belong to the same taxonomic category (Figure 1). After collecting these clades, the most inclusive ones are sought by analyzing nested relationships as follows: highly supported clades comprised of a single taxonomic category are grouped together, scaling back from the smallest clade to larger ones to find the most inclusive, or ‘largest’, clade which contains smaller clades, as long as the taxonomic composition remains the same. There can be two or more ‘largest’ clades for each taxonomic category. If the OTUs classified in a single taxonomic category are distributed across the tree in multiple highly supported clades, then multiple ‘large’ clades for that taxonomic category are recognized. For instance, if twenty *Homo sapiens* OTUs constitute three separate clades with 100% support values in a tree, and the user specifies the genus ‘*Homo’* as a taxonomic category to be pruned, TreeTrimmer recognizes three separate clades for the genus to be considered further.

For each of the largest highly supported clades, the branch lengths from the basal node to each leaf (i.e., the terminal node representing the OTU) are calculated and ranked in order of closeness to the median subtree branch lengths calculated using all the leaves within the clade. All the OTUs in the largest highly supported clades are then removed except for a specified number of OTUs possessing branch lengths best representing the median length (use of median values minimizes the impact of one or more unusually long-branching OTUs that deviate from the mean). TreeTrimmer returns two output files. The first, and most important, output is a list of OTUs retained after pruning, with information on how many OTUs have been removed in each of the highly supported clades. The second file is a trimmed tree (Figure 1B and 1C), which is used for reference (not analytical use), generated from the original tree file by removing nodes corresponding to the pruned OTUs. This trimmed reference tree lacks branch lengths and its topology is not necessarily phylogenetically meaningful; it is provided simply as a guide for downstream analyses.

## Findings

### Pruning redundant OTUs in single or multiple gene/protein trees

TreeTrimmer is a useful procedure for systematically and reproducibly pruning ‘redundant’ OTUs from phylogenetic trees and producing an alignment with fewer OTUs that are nevertheless representative of the original taxonomic diversity. Here ‘redundant’ OTUs refer to homologous sequences from related organisms belonging to the same taxonomic category with a level of taxonomic hierarchy (species, family, super-group, etc.) specified by the user in the parameter input file. Such redundant OTUs increase the quantity of data in an analysis but do not necessarily make a meaningful impact in terms of taxonomic diversity. Molecular phylogenetic studies are often focused on a particular lineage or set of lineages; additional OTUs are needed for the purposes of constructing and rooting the tree, but it is not necessarily important or practical to keep all such sequences.

We demonstrate the utility of TreeTrimmer by way of the simplified analysis of mitochondrial cytochrome c oxidase genes presented in Figure 1 (see Additional file 1: Figure S1 for the fully annotated tree). If the goal is to investigate phylogenetic relationships across the full breadth of eukaryotes, one may not want to include sequences from the full diversity of animals, whose genomes are extensively sequenced compared to most other lineages. In this case, the parameter input file is used to up-weight or down-weight different taxonomic categories by specifying how many OTUs should be retained after the dereplication procedure (compare Figure 1B and 1C). No OTUs are removed from clades with statistical support that falls below the chosen threshold. Also, the dereplication procedure ignores clades containing OTUs fewer than the set minimum number of the OTUs to be retained. For example, ‘slime mold’ is not subject to dereplication since the number of amoebozoan OTUs (in this case, one) is smaller than the minimum number assigned for Amoebozoa (two) in Figure 1C.

### User-defined taxon sampling with TreeTrimmer

As shown above, TreeTrimmer can be used to minimize the loss of taxonomic breadth when trimming down a phylogenetic dataset. The procedure represents an advance over the commonly used approach of simply taking the top-ranked hits from a similarity search, e.g., BLAST hits [3], since the similarity scores in such searches do not always capture phylogenetic relatedness between query and hit [8]. An analysis of photosystem II manganese-stabilizing protein (PsbO) proteins (Additional file 1: Figure S2A) (maximum number of BLASTP hits, 2000; e-value cut off, 1e-5) shows that use of TreeTrimmer results in a much-reduced dataset and, ultimately, a ‘second round’ tree composed of 75 OTUs (Additional file 1: Figure S2B) instead of 224 in the original (Additional file 1: Figure S2A). In terms of retention of taxonomic diversity, this result contrasts the nature of the dataset obtained simply by modifying BLAST-based sequence retrieval parameters. For example, use of a more stringent threshold value (1e-100) to eliminate low scoring sequences (Additional file 1: Figure S2C) or limiting the total number of sequences retrieved by BLASTP to 100 (Additional file 1: Figure S2D) resulted in second round trees with similar numbers of OTUs, but with only green plant sequences present (green fonts in Additional file 1: Figure S2). Clearly this is not useful if the goal is to produce a tree of PsbO proteins representative of the whole of plant/algal diversity. In sum, TreeTrimmer can reduce dataset size by selectively pruning OTUs from taxon-rich clades, resulting in alignments that are compatible and manageable with memory-intensive phylogenetic programs such as those employing Bayesian approaches [9,10].

### Streamlining paralogous gene families

Another useful application of TreeTrimmer is to mitigate the ‘paralogy problem’, i.e., inclusion of unnecessarily large numbers of paralogs from a single genome retrieved from automated similarity searches and assembled into multiple sequence alignments. Paralog redundancy can unnecessarily complicate interpretation of the tree topology and, for examining the relationships between higher order taxa, it is helpful to collapse the clades containing only redundant paralogs in highly duplicated genomes (e.g., closely related paralogs from the same species or the same group defined by users) into a number of representatives. Paralog reduction using TreeTrimmer is shown using the example of Myb-domain containing transcription factors found in six land plant genomes (Additional file 1: Figure S3A). In this example, 1016 OTUs from members of the Viridiplantae (green plants), including Bryophyta (mosses) and Tracheophyta (vascular plants), within a highly supported basal clade (SH value 0.977 shown with asterisk in Additional file 1: Figure S3A) was trimmed down to 5 OTUs (Additional file 1: Figure S3B). TreeTrimmer can also produce a less aggressively trimmed dataset with different parameter settings, e.g., by pruning only highly supported clades containing all Bryophyta or all Tracheophyta OTUs into 2 OTUs per clade, and retaining clades with support values less than 0.8, for second round tree construction (68 OTUs in total; Additional file 1: Figure S3C).

Given that phylogenetic trees are often biased taxonomically due in part to genome sequencing efforts being focused on model organisms and humans, one may want to employ an objective and reproducible method to minimize this bias. As of Oct 2, 2012, the NCBI taxonomy database (http://www.ncbi.nlm.nih.gov/taxonomy) contained 3,116 ‘species’ with ‘Genome’ accessions in the taxonomic category ‘Metazoa (metazoans)’, while the ‘Rhodophyta (red algae)’ category had just 19. There were also substantial differences in availability of sequence data among prokaryotic taxa, e.g., ‘Proteobacteria’ (2,467 species) was forty times more ‘accession-rich’ than ‘Crenarchaeota (eocytes)’, for which only 59 were deposited. TreeTrimmer provides a simple way to ‘optimize’ taxon sampling for a given phylogenetic analysis.

## Conclusions

Recent advances in genome and transcriptome sequencing have enabled taxon-rich sampling for single or multiple gene phylogenetic analyses. Nevertheless, it is not trivial to ensure balanced taxon sampling in an objective and reproducible way. We developed a tree-based dereplication method for pruning redundant OTUs from phylogenetic datasets based on support values, branch lengths and taxonomic information linked to each sequence. This method enables the user to reproducibly obtain reduced OTU datasets with user-defined parameters, yielding alignments amenable for use with a wider range of computer-intensive downstream analyses in automated or manual phylogenetic pipelines.

## Availability and requirements

TreeTrimmer script and sample data are available at the following Google Code site (http://code.google.com/p/treetrimmer/).

## Competing interests

The authors declare that they have no competing financial interests.

## Authors’ contributions

SM and RJME developed the algorithm and wrote the program. SM and JMA designed the study and drafted the manuscript. JMA conceived of and coordinated the study. All authors read and approved the final manuscript.

## Supplementary Material

Additional file 1: Figure S1 Phylogeny of Cytochrome c oxidase subunit 2 proteins. **A**) Phylogenetic tree of Cytochrome c oxidase subunit 2 proteins used in Figure 1A, with full descriptions of organismal names and accession numbers. Parameter input files (**B**) and (**C**) were used to generate the trees shown in Figure 1B and C, respectively, together with the Newick format input tree file (**D**) and the reference list of OTU names and taxonomic information (**E**). **Figure S2.** PsbO protein phylogeny with the query from *Arabidopsis thaliana* using various settings. Settings were as follows: (**A**) Maximum number of BLASTP hits retrieved, 2000; BLASTP cutoff value, 1e-5. Font colors represent taxonomic categories. (**B**) Dereplication cutoff, 0.8; the numbers of OTUs retained are 2 for Alveolata, 2 for Stramenopiles, 2 for Euglenozoa, 2 for Viridiplantae, and 2 for each genus if not included in these taxonomic categories. Note that the sequences were re-collected based on the TreeTrimmer output and re-aligned prior to constructing the tree. (**C)** Maximum number of BLASTP hits retrieved, 2000; BLASTP cutoff value, 1e-100. (**D**) Maximum number of BLASTP hits retrieved, 100; BLASTP cutoff value, 1e-5. **Figure S3.** Protein phylogeny of Myb-domain containing transcription factors from green plants (Viridiplantae). (**A**) Sequences homologous to GenBank accession BAA23337 (*Oryza sativa* OSMYB1) were collected from *Arabidopsis thaliana* (Tracheophyta), *Oryza sativa* (Tracheophyta), *Zea mays* (Tracheophyta), *Brachypodium distachyon* (Tracheophyta), *Vitis vinifera* (Tracheophyta), *Physcomitrella patens* (Bryophyta), and *Cyanidioschyzon merolae* (red alga, outgroup) by BLASTP, with the maximum number of hit 5000 and the e-value cut off 1e-5. Species names in Green, Tracheophyta; Blue, Bryophyta; Magenta, outgroup (red algal) OTUs. The support value for the whole Viridiplantae clade is shown in bold with an asterisk. (**B**) The tree was reconstructed using the TreeTrimmer output with the following settings: Support value cutoff, 0.8; the numbers of OTUs retained are 5 for Viridiplantae. (**C**) Tree was built in the same manner as in B but the numbers of OTUs retained are 2 for Bryophyta and 2 for Tracheophyta. Click here for file
