# Supplementary material for: Treetrimmer: a method for phylogenetic dataset size reduction
Source: BMC Res Notes. 2013 Apr 12;6:145. doi: 10.1186/1756-0500-6-145 (PMC3637088; doi:10.1186/1756-0500-6-145)
Supplement: Additional file 1: Figure S1 — Phylogeny of Cytochrome c oxidase subunit 2 proteins. A) Phylogenetic tree of Cytochrome c oxidase subunit 2 proteins used in Figure 1A, with full descriptions of organismal names and accession numbers. Parameter input files (B) and (C) were used to generate the trees shown in Figure 1B and C, respectively, together with the Newick format input tree file (D) and the reference list of OTU names and taxonomic information (E). Figure S2. PsbO protein phylogeny with the query from Arabidopsis thaliana using various settings. Settings were as follows: (A) Maximum number of BLASTP hits retrieved, 2000; BLASTP cutoff value, 1e-5. Font colors represent taxonomic categories. (B) Dereplication cutoff, 0.8; the numbers of OTUs retained are 2 for Alveolata, 2 for Stramenopiles, 2 for Euglenozoa, 2 for Viridiplantae, and 2 for each genus if not included in these taxonomic categories. Note that the sequences were re-collected based on the TreeTrimmer output and re-aligned prior to constructing the tree. (C) Maximum number of BLASTP hits retrieved, 2000; BLASTP cutoff value, 1e-100. (D) Maximum number of BLASTP hits retrieved, 100; BLASTP cutoff value, 1e-5. Figure S3. Protein phylogeny of Myb-domain containing transcription factors from green plants (Viridiplantae). (A) Sequences homologous to GenBank accession BAA23337 (Oryza sativa OSMYB1) were collected from Arabidopsis thaliana (Tracheophyta), Oryza sativa (Tracheophyta), Zea mays (Tracheophyta), Brachypodium distachyon (Tracheophyta), Vitis vinifera (Tracheophyta), Physcomitrella patens (Bryophyta), and Cyanidioschyzon merolae (red alga, outgroup) by BLASTP, with the maximum number of hit 5000 and the e-value cut off 1e-5. Species names in Green, Tracheophyta; Blue, Bryophyta; Magenta, outgroup (red algal) OTUs. The support value for the whole Viridiplantae clade is shown in bold with an asterisk. (B) The tree was reconstructed using the TreeTrimmer output with the following settings: Support value cutoff, 0.8; the numbers o [file 1756-0500-6-145-S1.pdf]

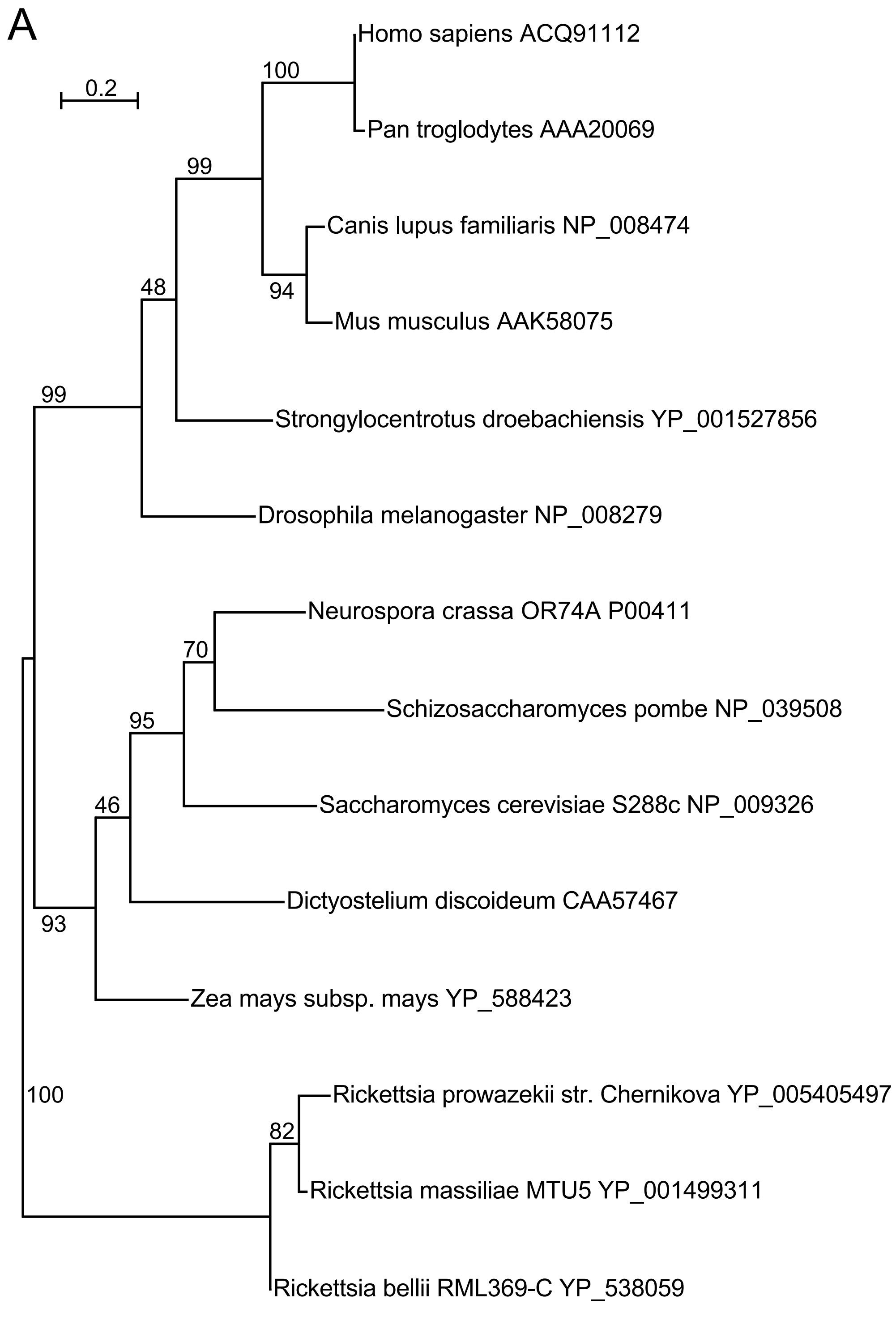

Fig. S1 Phylogeny of Cytochrome c oxidase subunit 2 proteins.

A) Phylogenetic tree of Cytochrome c oxidase subunit 2 proteins used for Fig. 1A, with full descriptions of organismal names and accession numbers. Parameter input files (B) and (C) were used to generate Fig. 1B and C, respectively, together with the Newick format input tree file (D) and the reference list of OTU names and taxonomic information (E).

B

```
# Which taxonomic categories should be pruned? How many OTUs should be retained?
Bacteria        2
Eukaryota       2

# Cutoff value for de-replication
cutoff=0.8

# How many OTUs should be retained in each clade unless specified above?
num_retained=2
```

C

```
# Which taxonomic categories should be pruned? How many OTUs should be retained?
Bacteria        2

Metazoa         2
Fungi   2
Viridiplantae   2

# Cutoff value for de-replication
cutoff=0.8

# How many OTUs should be retained in each clade unless specified above?
num_retained=2
```

D

```
(YP_538059:0.00017,(YP_001499311:0.01794,YP_005405497:0.07602)0.824:0.07449,((YP_58
8423:0.23983,(CAA57467:0.39789,(NP_009326:0.34317,(NP_039508:0.44084,P00411:0.23284)
0.709:0.08054)0.953:0.14077)0.468:0.09091)0.935:0.15749,(NP_008279:0.29288,(YP_0015278
56:0.25260,((AAK58075:0.06185,NP_008474:0.04192)0.948:0.11634,(AAA20069:0.02460,ACQ
91112:0.00016)1.000:0.23993)0.995:0.22741)0.480:0.08723)0.997:0.27967)1.000:0.68421);
```

E

```
ACQ91112        Eukaryota; Metazoa; Homo sapiens.
AAK58075        Eukaryota; Metazoa; Mus musculus.
AAA20069        Eukaryota; Metazoa; Pan troglodytes.
NP_008474       Eukaryota; Metazoa; Canis lupus familiaris.
NP_008279       Eukaryota; Metazoa; Drosophila melanogaster.
YP_001527856    Eukaryota; Metazoa; Strongylocentrotus droebachiensis.

NP_009326       Eukaryota; Fungi; Dikarya; Ascomycota; Saccharomyces cerevisiae S288c.
NP_039508       Eukaryota; Fungi; Dikarya; Ascomycota; Schizosaccharomyces pombe.
P00411  Eukaryota; Fungi; Dikarya; Ascomycota; Neurospora crassa OR74A.

CAA57467        Eukaryota; Amoebozoa; Mycetozoa; Dictyosteliida; Dictyostelium; Dictyostelium discoideum.

YP_588423       Eukaryota; Viridiplantae; Poaceae; Zea mays subsp. mays.

YP_538059       Bacteria; Proteobacteria; Rickettsiales; Rickettsia bellii

YP_005405497    Bacteria; Proteobacteria; Rickettsiales; Rickettsia prowazekii str. Chernikova.

YP_001499311    Bacteria; Proteobacteria; Rickettsiales; Rickettsia massiliae MTU5.
```

Fig. S1 Phylogeny of Cytochrome c oxidase subunit 2 proteins (continued).

Fig. S2A

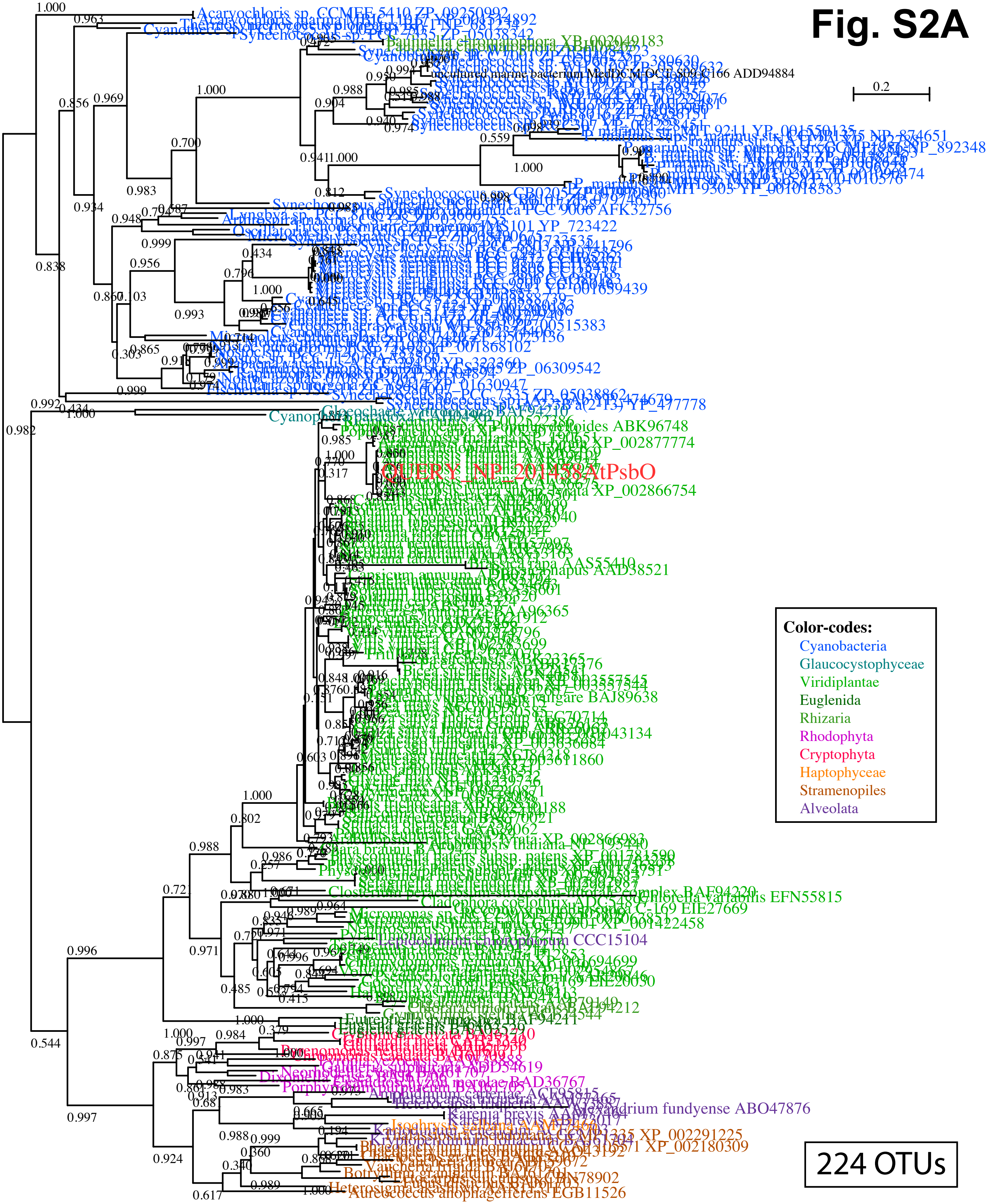

PsbO protein phylogeny with the query from Arabidopsis thaliana and the following settings:
Maximum number of BLASTP hits retrieved, 2000; BLASTP cutoff value, 1e-5.
Font colors represent taxonomic categories.

Fig. S2B

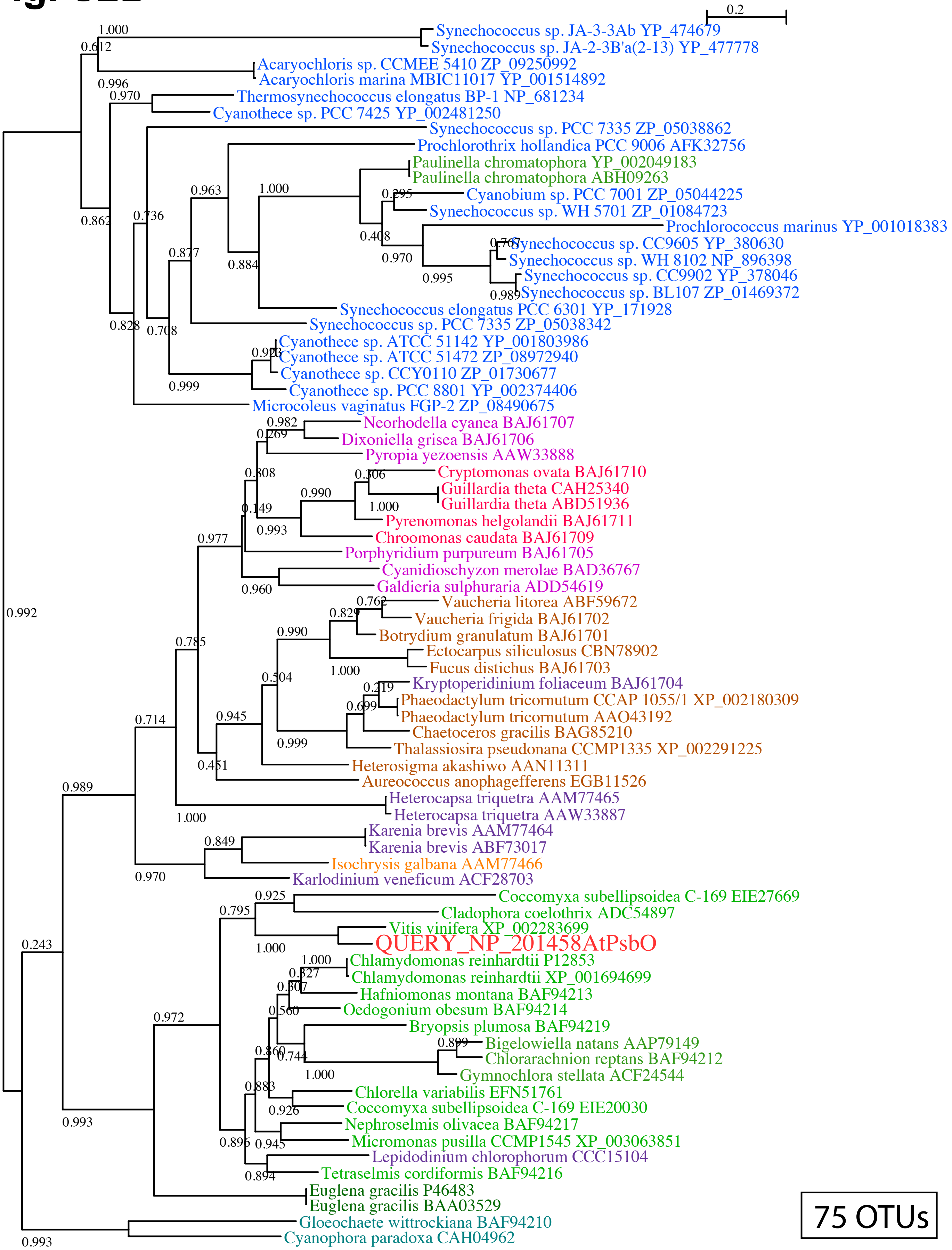

PsbO protein phylogeny with the query from Arabidopsis thaliana and the following settings: dereplication cutoff, 0.8; the numbers of OTUs retained are 5 for Bacteria, 5 for Archaea, 2 for Metazoa, 2 for Fungi, 2 for Alveolata, 2 for Stramenopiles, 2 for Euglenozoa, 2 for Viridiplantae, and 2 for each genus if not included in those taxonomic categories.

Fig. S2C

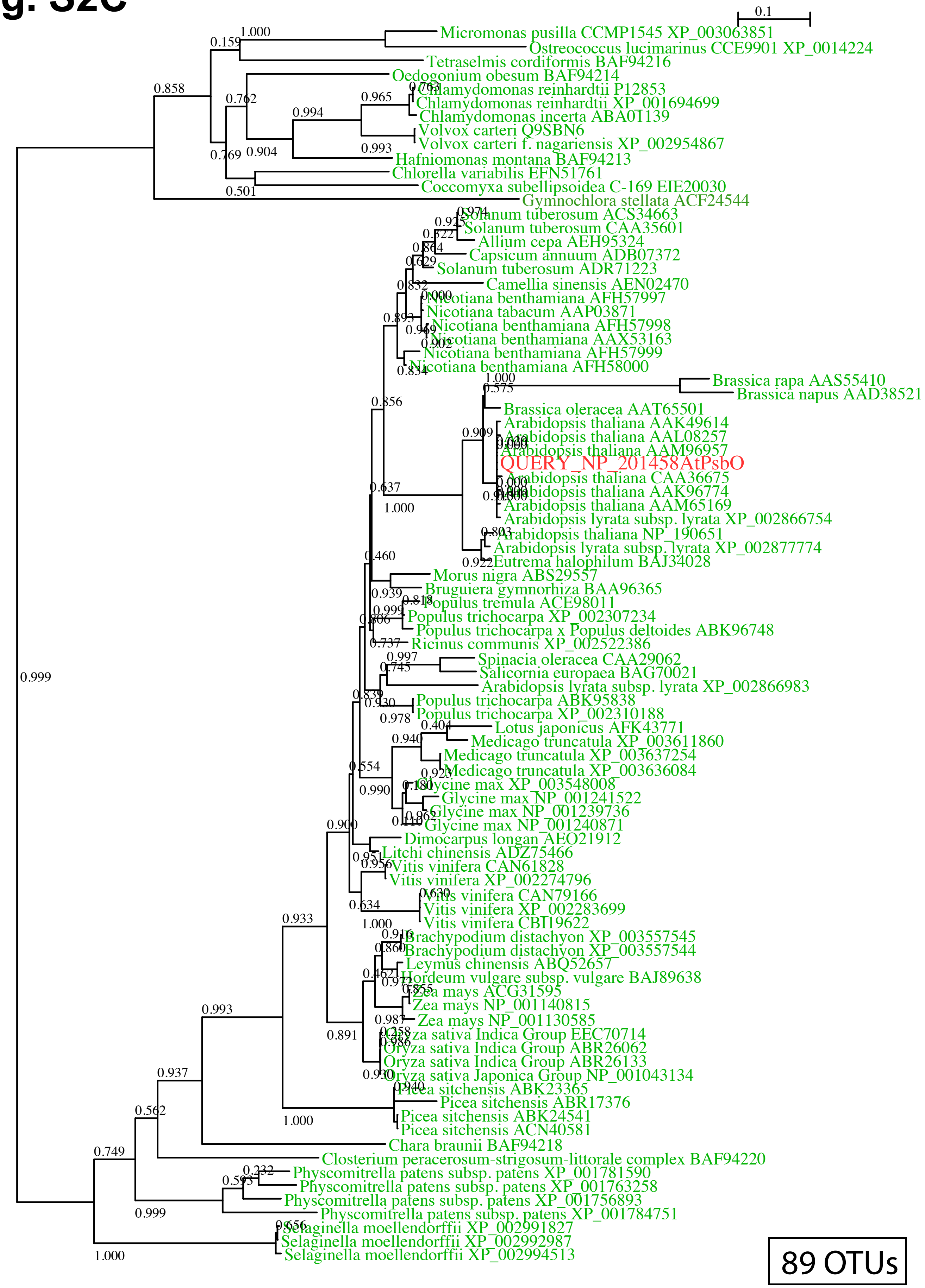

PsbO protein phylogeny with the query from Arabidopsis thaliana and the following settings: Maximum number of BLASTP hits retrieved, 2000; BLASTP cutoff value, 1e-100.

Fig. S2D

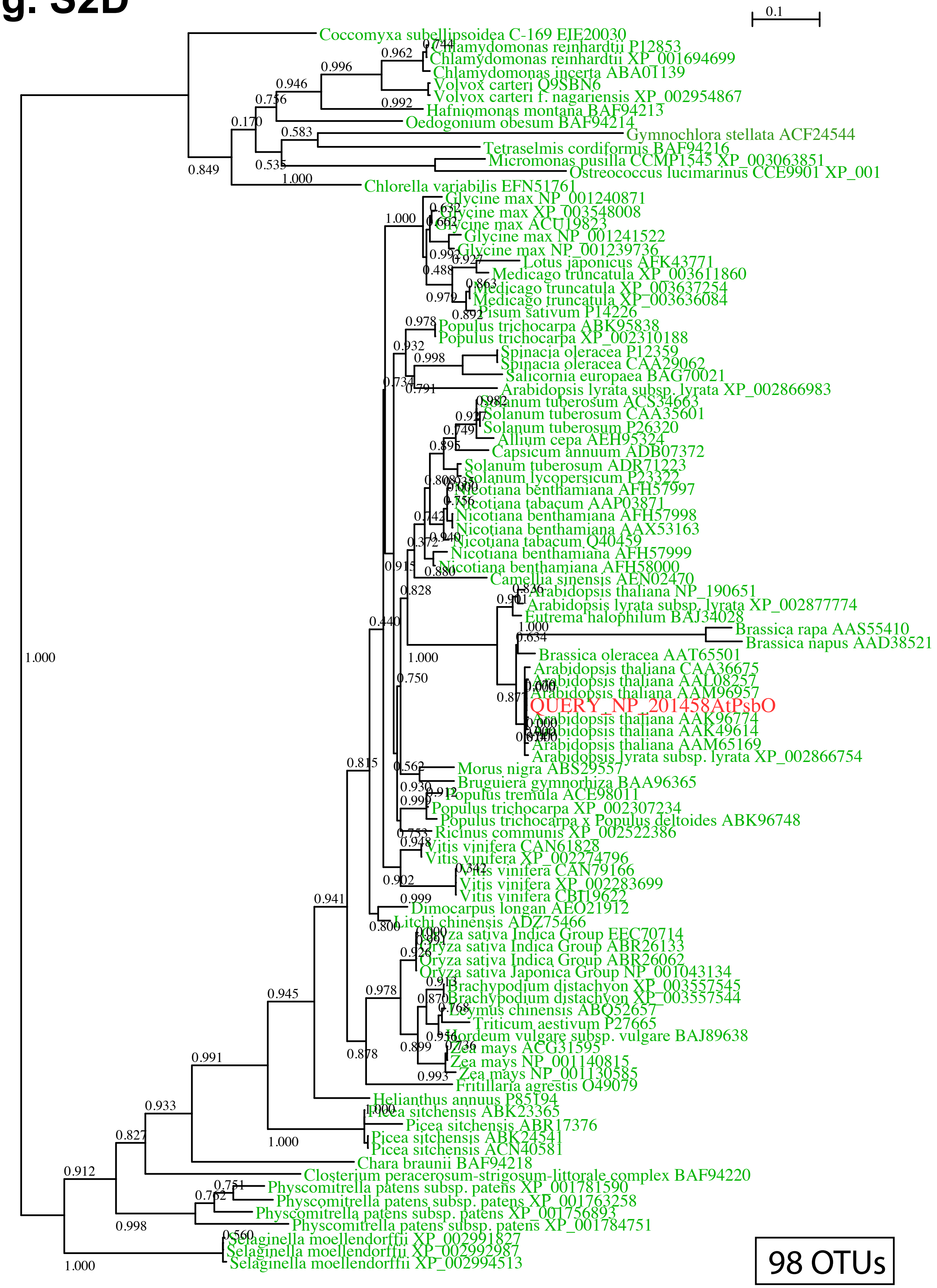

PsbO protein phylogeny with the query from Arabidopsis thaliana and the following settings: Maximum number of BLASTP hits retrieved, 100; BLASTP cutoff value, 1e-5.

Fig. S3A

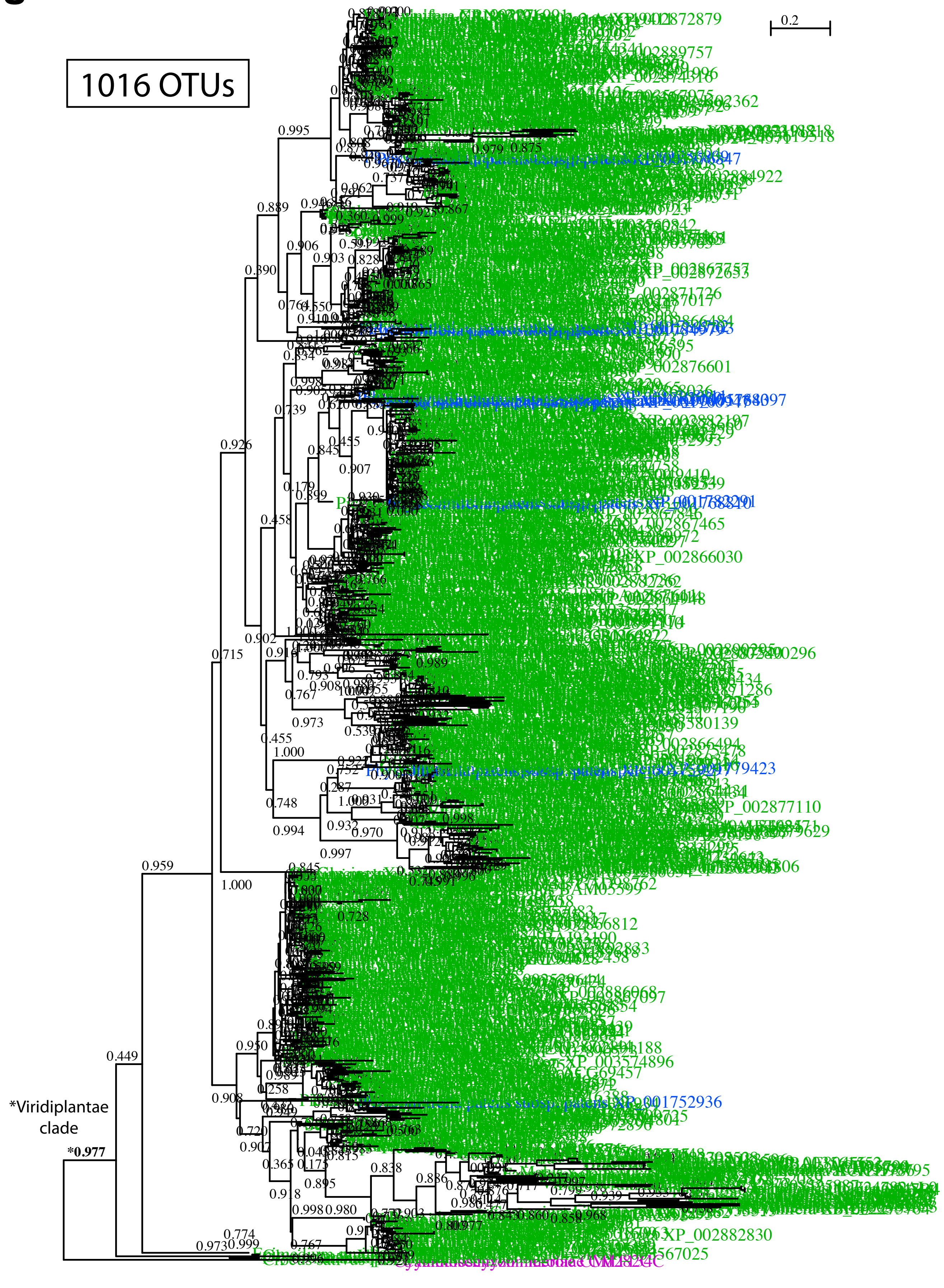

Protein phylogeny of Myb-domain containing transcription factors from green plants (Viridiplantae). Species names in Green, Tracheophyta; Blue, Bryophyta; Magenta, outgourp (red algal) OTUs.

Fig. S3B

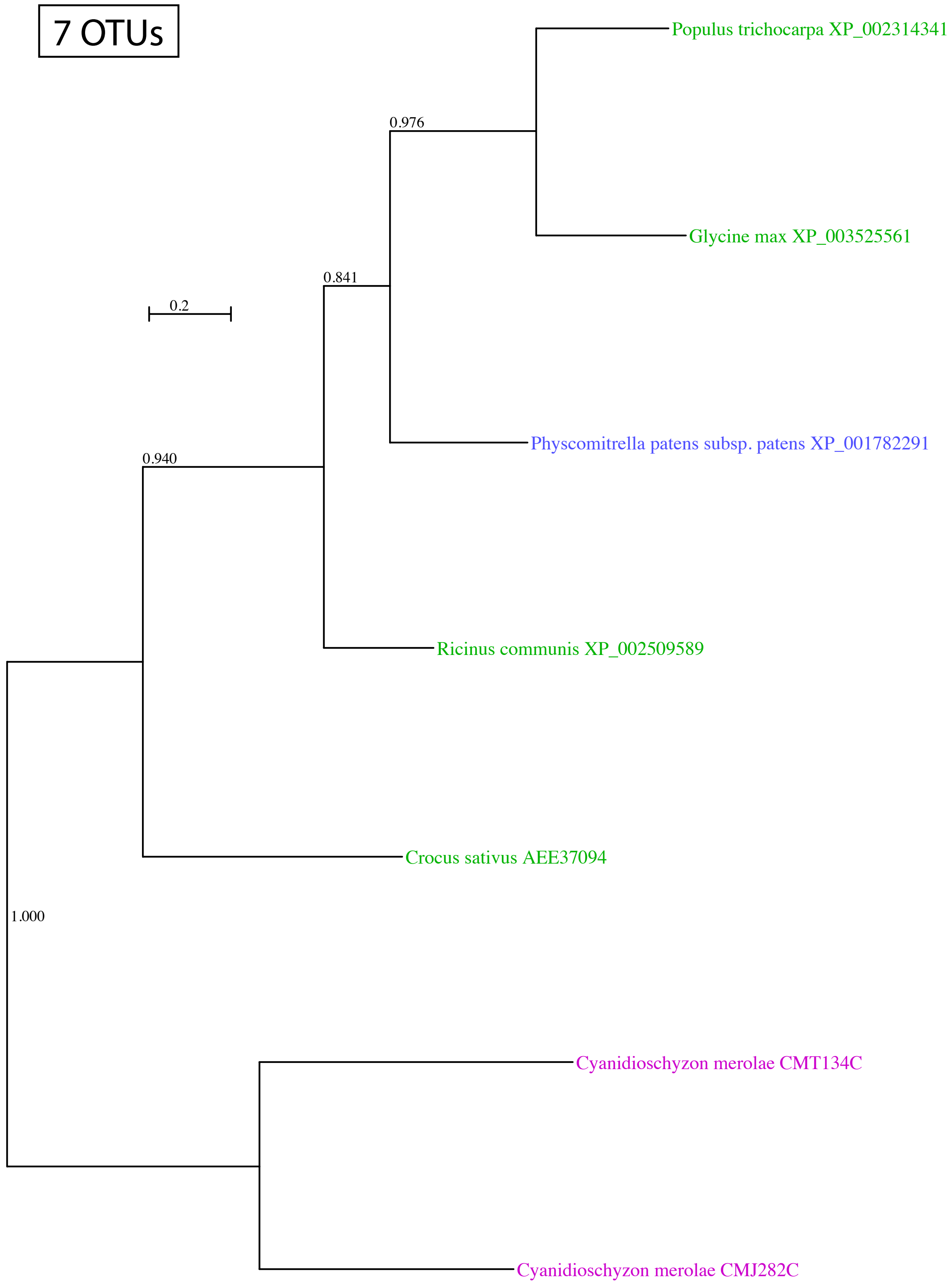

Reconstructed tree from TreeTrimmer output with the following settings: support value cutoff, 0.8; the numbers of OTUs retained are 5 for Viridiplantae.

Fig. S3C

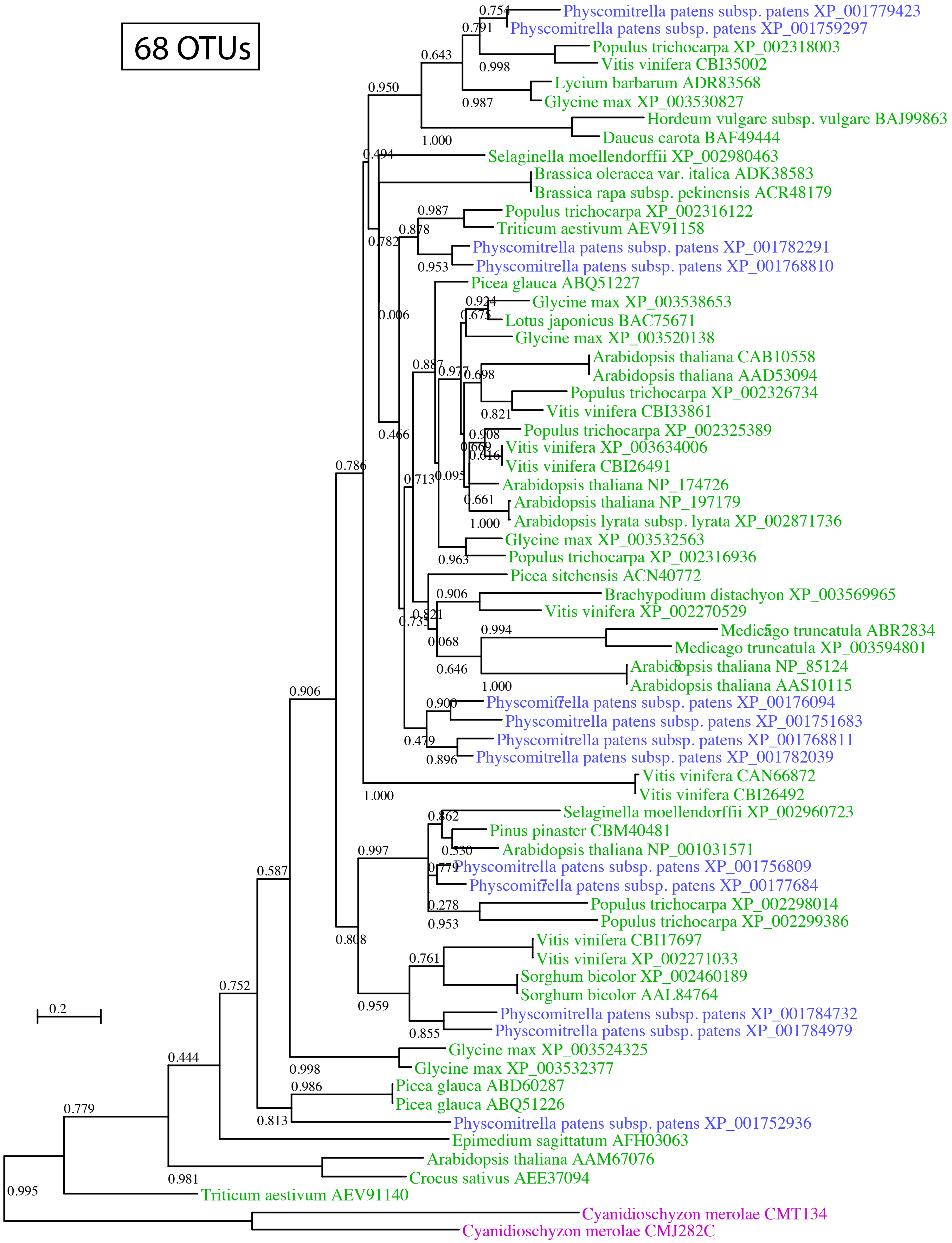

Reconstructed tree from TreeTrimmer output with the following settings:
support value cutoff, 0.8; the numbers of OTUs retained are 2 for Bryophyta; 2 for Tracheophyta.
